# Government Calling Revisited: A Survey-Experiment on the Moderating Role of Public Service Motivation in Assessing Employer Attractiveness

**DOI:** 10.3389/fpsyg.2020.559011

**Published:** 2020-10-29

**Authors:** Wouter Vandenabeele, Stefanie Jager

**Affiliations:** ^1^Utrecht School of Governance, Utrecht University, Utrecht, Netherlands; ^2^Public Governance Institute, KU Leuven, Leuven, Belgium

**Keywords:** public service motivation, employer attractiveness, person–organization fit, recruitment messages, public values

## Abstract

Employer attractiveness is an important variable for any organization. It is therefore not surprising that organizations try to control this facet when communicating recruitment messages for positions to be filled. This study aims to capture this process for public sector organizations, while looking at the role that a particular type of prosocial motivation – public service motivation: the motivation people have to contribute to society – plays in this process. To this end, a survey-experiment (*N* = 192) with prospective employees is carried out in which recruitment messages with three different value statements (public, private, neutral) are presented to the respondents. The effect of these message on both attractiveness and person–organization fit, as moderated by public service motivation, is tested. The results indicate that public service motivation indeed moderates the effect of these messages. However, the results do not fully corroborate the theoretical expectations. Therefore, additional exploratory analyses are performed in order to better understand the variables included in this process. This provides a direction for further research. Theoretical and practical implications are discussed.

## Introduction

The belief that organizational processes can only be successful if they capitalize on rational and self-interested motives has been long brushed aside and “economic man” – an individual steered by rational cognitive processes – has long been declared dead (Simon, 1997). Furthermore, other motives with other orientations have been highlighted in various fields of the social sciences. For example, within nursing, compassion is seen as an important attribute (Peters, 2018), environmental sciences talk about sustainability as a characteristic (Presley et al., 2018), while the management literature talks about corporate social responsibility as an important driver (Belinda et al., 2018). The common thread in these observations can be conveniently captured under the heading of prosocial motivation and the related prosocial behavior (Bolino and Grant, 2016). Public service motivation is a particular type of prosocial motivation that is seen as especially relevant within the field of public administration. It is almost 40 years since the term was coined by Hal Rainey (1982), and 30 years since it was formally conceptualized by Perry and Wise (1990), and it remains a thriving and vibrant concept in this field (Ritz et al., 2016).

Since its first appearance, numerous studies have been carried on this particular concept (Vandenabeele et al., 2014; Ritz et al., 2016). However, despite its prominence, at least one topic has not been widely addressed. This void in the literature concerns the translation of knowledge on public service motivation (PSM) into practical applications and assessing the value of these applications. Paarlberg et al. (2008) were already indicating that a crucial element was translating this public service motivation into practice. To do so, they offered five, sometimes overlapping, domains in which public service motivation could be utilized to benefit the provision of public service. These domains are its inclusion in basic HRM processes, including job characteristics that appeal to public service motivated employees, developing a work environment that is public service motivation friendly, including public service based values in the mission, and fostering public service motivation outside of the organization. Despite this, only a few studies have transposed knowledge to practical applications (Christensen et al., 2017) and even fewer studies have integrated them in general human resources management (HRM) models (Vandenabeele et al., 2013). Very few have incorporated prosocial elements as a focus in strategic HRM models despite the widespread acknowledgment that a best-fit approach in which the HR strategy is aligned with strategic elements of the organizational strategy is a particularly successful one (Boxall and Purcell, 2016). As a consequence, very little of the current knowledge on prosocial or public service motivation has actually been put to work.

Notable exceptions in this respect include studies regarding recruitment and selection (Asseburg et al., 2018; Weske et al., 2019) that relate to the first two domains of Paarlberg et al. (2008). These studies provide mixed results in terms of the value of public service motivation. Whereas Weske et al. (2019) did not find any significant effect of considering public service motivation in recruiting potential employees, the study by Asseburg et al. (2018) did find positive effects of public service motivation, in particular on the extent of communicating about tasks, which was moderated by public service motivation. Another study (Linos, 2018) provided mixed evidence. These findings therefore justify more research given the inconsistent data. Replicating research findings, be it in terms of strong replications that exactly replicate research designs, or weak replications seeking general effects (creating an opportunity to include context and moderators), is necessary to determine meta-effects and better assess the “true effects” (Ioannidis, 2005).

Furthermore, although a recent meta-analysis has indicated that public service motivation is important for public sector recruitment (Asseburg and Homberg, 2020), previous studies – apart from the above – that suggest effectiveness suffers from a general lack of convincing causal claims. More recent studies by Christensen and Wright (2011); Ballart and Rico (2018), and Asseburg and Homberg (2020) as well as earlier work on employer attractiveness (Lewis and Frank, 2002; Mann, 2006; Vandenabeele, 2008a) also raise concerns regarding causal inference. Causal claims might be better demonstrated through an experimental design in order to satisfy the conditions for demonstrating causality (Kenny, 2004; Antonakis et al., 2010).

In response to these concerns, the present study aims to contribute to the literature in two ways. First, by addressing employer attractiveness, in particular as a practical contribution to recruitment practices in the public sector, and in other sectors where prosocial values play an important role within the organizational strategy. Second, by investigating the role of public service motivation in relation to person–organization fit, we provide evidence of the relationship between public service motivation and a more general approach to HRM. This concept of fit is not only crucial for contextualizing HRM, but also for creating positive HRM outcomes (Boon et al., 2011). These objectives are addressed through a survey-experiment, an approach that can capitalize on the strengths of an experiment through randomization (Jilke and Van Ryzin, 2017). The survey sample consists of potential employees of a public organization – in this case a municipality – as all the participants are active on the labor market. In particular, the research tries to provide an answer to the question:

To what extent does public value-based content in recruitment messages influence actual organizational attractiveness and perceived person–organization fit of a public employer and what is the role of public service motivation in this process?

### Employer Attractiveness

The attractiveness of an organization to prospective employees is a vital characteristic in meeting that organization’s strategic human resource management objective, and hence in being successful. First, attractiveness is crucial for any organization to be able to attract well-qualified and motived candidates for vacancies who will create added value from a human resources perspective (Leisink and Steijn, 2008). Attractiveness is most likely the best precursor of success in this process since attitudes are the best predictor of (hypothetical future) behavior. Therefore, the attractiveness of an organization – an individual being attracted to the organization – would be the first, and perhaps most important, step in the process of being recruited by an organization if we look at this process from the perspective of the Attraction-Selection-Attrition (ASA) model (Schneider, 1987).

Attractiveness can be viewed from two similar, but not entirely overlapping, perspectives, namely the organization versus the employment perspective. Looking at it from the perspective of an organization, Ritz and Waldner (2011) conceive employer attractiveness as “the interest of an individual to work within a certain organization” (p. 293), as it had been defined earlier by Lieber (1995). This interest refers to the entire organization, and not to one particular job within it. A similar position is found in Highhouse et al. (2003), who also refer to the process of attraction, but stress even more its individual nature by using the concept of attraction – seen as an individual-level construct – as opposed to the term attractiveness which is more of an organizational characteristic. It is defined accordingly as “individuals’ affective and attitudinal thoughts about particular [organizations] as potential places for employment” (p. 989). This is closer to an attitude and therefore fits better with the ASA theories mentioned above.

Alternatively, one could look at attractiveness from an employment perspective, as for example by Berthon et al. (2005). However, such definitions relate more to “the envisioned benefits that a potential employee sees in working for a specific organization” (Kashive and Khanna, 2017, p. 156) and are therefore referring more to antecedents of attractiveness (or the process of attractiveness) than to the concept itself.

Our study uses the term attractiveness, rather than attraction, because we see it as important to conceive the object of the study as an individual’s idiosyncratic perception of an organization’s attributes, indeed the entire organization forming an external object, that cause attraction to that organization. The definition by Highhouse et al. (2003) is applied as it so elegantly places the construct within the individual. Moreover, this definition also provides a clear pointer to what it actually is: an individual-level attitude.

### Public Service Motivation: A Particular Type of Prosocial Motivation

Prosocial motivation is a broad concept and the study of prosociality includes many aspects. First, it is important to distinguish between prosocial motivation and prosocial behavior, with the latter being an outcome of the former. The concept of prosocial motivation can be defined as “the desire to expend effort to benefit other people” (Grant, 2008, p. 48). It may, but not necessarily, also encompass self-interested dimensions such as purely altruistic motives, which illustrates that there are many interpretations of the construct (Bolino and Grant, 2016; Böckler et al., 2016). Public service motivation, referring to a person’s motivation to contribute to society, is but one type of prosocial motivation and it can be distinguished from more general types. First, it is important to distinguish the target audience, as other types of prosocial motivation aim at a direct or reciprocal relationship with the beneficiary providing direct and identifiable benefits, whereas public service motivation does not involve this feedback mechanism since the beneficiary is unidentified (Vandenabeele et al., 2018; Schott et al., 2019). This results in a much more distant relationship with the beneficiary in terms space, time, and social relationship compared to other types of prosocial motivation (Ritz et al., 2020). Similarly, public service motivation is distinct from intrinsic motivation, although they can be strongly related and indeed some scholars define it is as a specific form of intrinsic motivation (Houston, 2000; Steijn, 2008). However, the idea of enjoyment that is inherent to intrinsic motivation (Ryan and Deci, 2004), is not a necessary component of public service motivation, as public service motivation leans heavily on the idea of public values being realized rather than enjoyment being gained (Houston, 2011; Kim et al., 2013). These considerations lead us to view public service motivation as a concept in its own right, one that is related to, but distinct from, other types of prosocial or intrinsic motivation (Vandenabeele et al., 2018; Schott et al., 2019; Ritz et al., 2020).

When delving into the specifics of public service motivation, one notes that it refers to the idea that some people have a need or a drive “to do good for others and shape the well-being of society” (Perry and Hondeghem, 2008). Although this idea had been around for a long time (Horton, 2008), it was not until Perry and Wise (1990) defined public service motivation as “an individual’s predisposition to respond to motives grounded primarily or uniquely in public institutions” (p. 368), that it became a concept in its own right. Some authors have developed their own definitions (Brewer and Selden, 1998; Rainey and Steinbauer, 1999; Vandenabeele, 2007), and some, mostly non-American, authors do not use the term at all when studying public service motivated behavior (Pratchett and Wingfield, 1996; Chanlat, 2003).

Despite the fact that in the first illustration of public service motivation it was addressed as an aid to alleviate “a quiet crisis in the federal civil service” (Perry and Wise, 1990, p. 367), the theory did not explicitly place the concept within the boundaries of the public sector. Although some confusion has existed over the years as to whether public service motivation could be applied to organizations and employees outside the narrow scope of the civil service, nowadays there is a broad consensus that it can. Theoretical developments have clarified that the use of public service motivation is not limited to the civil service, but rather to “public institutions organizations” (Perry and Wise, 1990, p. 368), as “it is in the public content of institutions in which public service motivation has its origins” (Perry and Vandenabeele, 2008, p. 60). Furthermore, empirical studies have placed public service motivation in various environments including beyond the civil service and the public sector in the strictest sense.

When it was first operationalized, Perry (1996) identified four dimensions of public service motivation: “Attraction to politics and policy making,” “Public interest,” “Compassion,” and “Self-sacrifice.” Later research (Camilleri, 2006, 2007; Bright, 2007; Coursey and Pandey, 2007; Coursey et al., 2008; Vandenabeele, 2008a,b; Kim, 2009a,b) have generally supported this factor structure, although in some cases subtle or more marked differences can be seen. More recent studies have reframed the concept in such a way that commitment to public values is more prominent as a dimension of public service motivation (Kim and Vandenabeele, 2010; Kim et al., 2013).

Notwithstanding the above suggestion that public service motivation is not necessarily related to a particular institutional context, it has always been linked to a preference for certain employers. Early studies identified either a moderate (Vandenabeele et al., 2004; Vandenabeele, 2008a) or weak (Lewis and Frank, 2002) relationship between public service motivation and the preference to work for public employers. More recent studies have provided more mixed evidence, with many finding a similar effect (Christensen and Wright, 2011; Piatak, 2016; Wright et al., 2017; Asseburg et al., 2018; Ballart and Rico, 2018; Asseburg and Homberg, 2020) but others not (Lee and Choi, 2016; Weske et al., 2019). A recent meta-analysis (Asseburg and Homberg, 2020 confirmed a dominant pattern of public service motivation being an antecedent of public employment (with the caveat that most of the evidence did not allow causal inference). The varying effect sizes suggest that there could be potential moderators that interact with this main effect. Here, Vandenabeele (2008a) suggests that the level of person–organization fit, based on the degree of publicness (Antonsen and Jørgensen, 1997), could be a cause of the varying effect sizes with respect to the relationship between public service motivation and the attractiveness of a public organization.

### Interaction With the Environment: Institutional Theory and Fit Theories in Public Service Motivation

Behavior that is based on public service motivation is often described and explained using institutional theory (Perry, 2000; Vandenabeele, 2007; Perry and Vandenabeele, 2008). Institutions are viewed as “a formal or informal, structural, societal or political phenomenon that transcends the individual level, that is based on more or less common values, has a certain degree of stability and influences behavior” (Peters, 2000). The common values in the particular kind of public institutions that Perry and Wise (1990) were referring to relate to the idea of publicness (Antonsen and Jørgensen, 1997) or to the idea of public values (Jørgensen and Bozeman, 2007) as values that illustrate a consensus as to how society should be organized (Bozeman, 2007). These public institutions could be situated on the macro-, meso-, or micro-levels depending on the degree of personal and direct interaction (Vandenabeele et al., 2014). Nevertheless, as explained below, the bulk will be on the organizational, meso-level.

Members of these institutions, or indeed prospective members, adhere to a logic of consequence whenever they act within the boundaries of such institutions (March and Olsen, 1989, 1995). This is why fire officers run into a burning building, despite the dangers to themselves, and why teachers attend unpaid staff meetings with colleagues in the evening or even at the weekend. They do so because they are supposed to do it within particular institutional boundaries (and they might not do so in other situations). The explanation of this logic of appropriateness lies within the conditional mechanism of person–environment fit. The interaction between the values of the individual’s institutional identity – in this case public service motivation as an individual level variable – and the institutional environment – which is based on public values and reflects these in all its institutional processes and products – creates a fit between the individual and the environment that influences behavior (Kristof-Brown et al., 2005). This “person–environment” fit can have many guises including person–organization fit, person–job fit, person–team fit, and person–supervisor fit.

Of these, the person–organization fit is used to explain organizational attractiveness within the public sector (Vandenabeele, 2008a; Ritz and Waldner, 2011). Person–organization fit has been defined as “the congruence between and the compatibility between people and organizations that occurs when: (a) at least one entity provides what the other needs, or (b) they share similar fundamental characteristics, or (c) both” (Kristof, 1996, pp. 4–5). The mechanism that explains the effect of such a fit lies largely in a supplementary fit (Kristof, 1996) that creates a sense of belonging and recognition when operating within an institution. Further, there is also an effect of the individual complementing (Kristof, 1996) the institution by providing what the organization needs (demands-abilities), and vice-versa – the institution providing what is needed or important for the individual (needs-supplies).

In a public sector context, public service motivation has long been considered an important element in achieving person–organization fit (Bright, 2007; Vandenabeele, 2007). Steijn even coined the term PSM-fit (2008) to emphasize the importance of PSM in the overall person–environment fit (and hence person–organization fit). The relationship between public service motivation and person–organization fit in a public sector environment has since been demonstrated numerous times in various contexts and settings (Bright, 2008; Liu et al., 2010; Christensen and Wright, 2011; Kim, 2012; Teo et al., 2016; Van Loon et al., 2017). Often, this has been addressed from a causal chain perspective with person–organization fit acting as a mediator. However, given the cross-sectional nature of most studies, these causal claims are somewhat tenuous. Further, given the strong relationship between person–organization fit and several beneficial outcome variables such as performance, satisfaction, and commitment (Kristof-Brown et al., 2005), it may be worthwhile to consider person–organization fit as an important variable in its own right and as an indicator of the quality of an employment situation. As such, it could serve as a more general variable in an HR-value chain (Boon et al., 2011). Such a focal variable could serve to indicate the health of an HR system. Much as early coal miners used a canary to indicate the quality of the air in the mine they were working in, high levels of person–organization fit could point to an effective and healthy system of HRM.

Despite the consistent, albeit to varying extents, relationship between public service motivation and person–organization fit, the public sector is not a single organization, or even made up of a set of uniform organizations. Rather, it is an “amalgam of organizations and institutions that provide public service in its broadest sense, a patchwork of organizations” (Vandenabeele, 2008a, pp. 1091–1092). That is, it is a set of institutions, interrelated at the organizational level, which all have strong ties with encompassing higher-level institutions (Scott, 2013). Consequently, organizational attributes within the public sector can vary between organizations. This means that the effects of fit do not only play a role at the sector level, but also to a large extent at the organizational level in distinguishing between various public organizations.

### Recruitment Information

Information about particular organizational attributes is intentionally (and sometimes unintentionally) communicated to non-members through recruitment material. When recruiting, organizations evidently want to communicate as much information as possible in order to attract prospective employees. If organizations are seen as more attractive, the likelihood of prospective employees actual applying increases (Gomes and Neves, 2011). In recruitment, communications are increasingly focused on “value-based recruitment” (Paauwe, 2007), as values are considered to be a crucial element in this communication.

Although it may appear self-evident that public organizations would aim to appeal to prospective employees by focusing on institutional characteristics (in this case values) that can lead to a person–organization fit, practice illustrates that this has not been widely implemented (Waldner, 2012). Nevertheless, some studies have investigated the effect on prospective employees of recruitment texts that appeal to public service motivation. Weske et al. (2019) found an effect of private sector values included in an organizational description interacting with the extrinsic motivation of individuals. However, they did not find a similar effect of public values. Asseburg et al. (2018) did however find that public service motivation moderated the effect of affective and normative information included in recruitment messages. Linos (2018) also illustrated that the promise of making a difference for one’s community could convince people to apply for police jobs, whereas the promise of serving others did not. However, based on a combination of theory and empirical findings, it seems likely that the relationship between certain value types and attractiveness is moderated by public service motivation. On this basis our first hypothesis, H1, is formulated as two sub-hypotheses:

H1A Applicants with high levels of PSM will judge an organization to more attractive if public values are highlighted in recruitment texts, whereas applicants with lower levels of public service motivation will judge these organizations to be less attractive.
H1B Applicants with high levels of PSM will judge an organization to be less attractive if non-public values are present in recruitment texts, whereas applicants with lower levels of public service motivation will judge these organizations to be more attractive.

Likewise, given the strong theoretical justifications for a relationship between public service motivation and person–organization fit, H2 is formulated as follows:

H2A Applicants with high levels of public service motivation will achieve higher levels of person–environment fit than applicants with lower levels of PSM in organizations where public values are present in recruitment texts.
H2B Applicants with high levels of public service motivation will achieve lower levels of person–environment fit than applicants with lower levels of PSM in organizations where non-public values are highlighted in recruitment texts.

## Materials and Methods

This section describes and discusses the design and methods that are used to test the hypotheses. First, the design and the data are discussed, followed by an overview of the measures that have been used to capture the concepts at hand. Finally, a brief discussion of the methods to analyze the results is provided.

### Data and Design

In order to better approximate the conditions to establish causal inference (Kenny, 2004), this study uses an experimental design embedded within a survey. Many types of experimental designs – with their own labels and characteristics – exist in survey research and stem from various traditions. One can sometimes distinguish overlapping labels such as split-ballot designs, policy capturing designs, (factorial) vignette studies, or conjoint experiments (Atzmüller and Steiner, 2010; Aguinis and Bradley, 2014; Steiner et al., 2016; Jilke and Van Ryzin, 2017). A common factor in all these type of designs (apart from the split-ballot design) is that they use vignettes in which the independent variable is manipulated to assess its effects. These vignettes are presented randomly to participants and this random characteristic enables the design to control for spurious effects (De Vaus, 2001). In this study, the vignettes manipulate the presence of public versus private values in the recruitment texts. A control group was also constructed in which value statements were not provided. In developing these vignettes, we tried to maintain a close link to actual job advertisements to increase ecological validity (Morton and Williams, 2010). Afterward, we discussed these with actual HR professionals. Each respondent was presented with a single vignette. Such a between-subjects design was appropriate as only one variable – the values highlighted in the recruitment information – was manipulated. As such, there was no need to present more than one vignette to each respondent. This approach resembles the design applied by Weske et al. (2019).

The survey in which the experiment was embedded was presented to respondents who were all active participants in the labor market. Subjects were identified through two routes. The first group consisted of alumni of a master program in public administration, and the second group were employees working for a municipality. Both groups resided in the Netherlands. In this respect, the study distinguishes itself from earlier work which has mainly used students (Asseburg et al., 2018; Weske et al., 2019). As participants who have actual labor market experience (as opposed to students) may have more varied needs, this approach may provide higher levels of external validity. Response rates from the two groups were respectively 35% and 7%, resulting in an adequate sample size of 192. The respondents average year of birth was 1980, with the oldest being born in 1952 and the youngest in 1996. The gender distribution was 43% male and 57% female. Of the participants, 93% had a master-level education. A series of simple t-tests revealed that both groups did not significantly differ on the moderator. Given that the independent variable was randomly assigned, no equivalent test was performed for the independent variable. The resulting Folded F was 1.02 (df1 = 151 and df2 = 39; *p* = 0.97), thereby rejecting the assumption of unequal variances, and a t-test showed that the difference in PSM between the two groups (−0.07) was not significant (*t* = −0.60; df = 190; *p* = 0.55).

The vignettes were originally presented randomly to 262 participants who had agreed to participate and had opened the online survey. However, not all the participants completed the survey to a sufficient level to be included in the dataset. Of the 192 who were included, 72 had been given the public-values vignette, 58 the private-values condition, and 62 the neutral control group text (see Table 1). Two variables were used to create a manipulation check to see whether respondents had read the vignettes thoroughly, maximizing the effect of the treatment. The first one asked the subjects to name the department of the municipality where the vacancy was placed. This information had been provided at the end of the vignettes. Only four of the 192 failed to answer correctly. The other variable was an objective measure of the time spent on the survey (as recorded by the survey platform Qualtrics). For the set of complete responses (*N* = 192), the median time spent was 316 seconds, whereas for the dropouts the median was 55 s. Given the median time spent on the survey, and that the four who had incorrectly identified the department had all spent well above the lower quartile of time spent (206 s), it was decided that all the responders who had completed the survey had been sufficiently exposed to the manipulation.

**TABLE 1:** Descriptives by type of vignette.

	N	Mean	SD	Min.	Max.
**PSM**					
Public	72	4.149	0.582	2.500	5.000
Private	58	4.034	0.708	2.000	5.000
Control	62	4.246	0.625	2.000	5.000
**Attractiveness**					
Public	72	3.528	0.704	1.500	5.000
Private	60	3.688	0.734	1.750	5.000
Control	63	3.722	0.662	2.250	5.000
**PO-fit**					
Public	72	3.431	0.739	1.667	5.000
Private	59	3.525	0.604	2.000	5.000
Control	62	3.349	0.564	1.667	5.000

### Measures

The independent variable that was manipulated was the type of values held by the institution, in this case a municipality, as communicated in the recruitment message and in particular the values of the department and the general work environment. As suggested by Waldner (2012), cues toward both the organization and the job were included. Given the strong overlap between person–organization fit and person–job fit (Van Loon et al., 2017), it was assumed that job cues would also affect person–organization fit. In addition to the vignette that was designed to include mainly public values, two others were designed with private values and neutral values respectively. The public condition was based on values that reflect the dimensions of the public service motivation measure developed by Kim et al. (2013), whereas the cues that reflected private (non-public) values were based on the work of Van der Wal et al. (2008), who had identified public, private, and neutral values prevalent in the Netherlands. More details of our three vignettes can be found in the Appendix.

Three constructs were measured using aggregated Likert-type scales (see Table 2), with possible answers ranging from 1 (totally disagree) to 5 (totally agree). The measure of organizational attractiveness was based on Highhouse et al. (2003) general dimension of organizational attractiveness. The original instrument had two other dimensions, prestige and intention to obtain/accept a job. However, compared to these two dimensions, the general dimension had higher factor loadings (and thus demonstrated the highest reliability and average variance extracted) in their research. Therefore, we opted to only use this dimension, especially since it also correlated substantially with the other dimensions. As direct measures have higher average correlations with organizational attractiveness than indirect measures (Kristof-Brown et al., 2005), we decided to select a direct measure for the operationalization of person–organization fit. This was based on the measure by Cable and Judge (1996) and included three items that directly and subjectively measure person–organization fit (as opposed to indirect measures that quantify the fit by combining individual and organizational information). Public service motivation was assessed based on a one-dimensional global measure developed by Vandenabeele and Penning de Vries (2015). This was because multidimensional measures of public service motivation often suffer from confounding effects due to their high inter-factor correlation. This choice of a composite or global measure has a number of benefits. First, this avoids dimensionality concerns (because of its global character) but can have equal explanatory power. For example, Vandenabeele and Penning de Vries (2015) showed that their measure, which we adopted, has equal explanatory power as the Kim et al. (2013) measure with four dimensions. Second, given that the adopted measure only has four items, as opposed to longer multidimensional measures where those validated in a European context have between 16 and 18 items (Perry, 1996; Vandenabeele, 2008b; Giauque et al., 2011; Kim et al., 2013), it places less time demands on those completing the survey and potentially results in a lower drop-out rate.

**TABLE 2:** Measurement model of variables (fully standardized).

		λ	AVE	Weighted Ω
**Organizational Attractiveness**				
Q3_1_1	I think this organization is a good place to work	0.78***	0.61	0.89
Q3_1_2	I would not be interested in working for this organization, unless as a last resort	NA		
Q3_1_3	This organization is an attractive employer to me	0.83***		
Q3_1_4	I would like to know more about this organization as a possible employer	0.58***		
Q3_1_5	A job with this organization is attractive to me	0.89***		
**Person–Organization Fit**				
Q3_1_6	My values match those of this organization	0.89***	0.70	0.88
Q3_1_7	I think my values fit those of this organization’s employees	0.78***		
Q3_1_8	The values and personality of this organization reflect my own values and personality	0.83***		
**Public Service Motivation**				
Q4_1_1	I am very motivated to contribute to society	0.91***	0.69	0.93
Q4_1_2	Being able to contribute to society is very motivating	0.85***		
Q4_1_3	Making a difference in society, no matter how small, is very important to me	0.58***		
Q4_1_4	Defending the public interest is very important to me	0.93***		

*** *p* < 001.

The measures used have been validated by means of confirmatory factor analysis (LISREL 8.80) using a DWLS estimation to take account of the ordinal nature of the data (Jöreskog and Sörbom, 2004). As the data collected included a substantial number of missing values, the respondents with missing values were automatically removed from the analysis by LISREL, resulting in an effective sample of *N* = 192. On the basis of the modification indices and theoretical considerations, the model was re-specified once, removing one item on empirical and theoretical grounds, to obtain a measurement model of the three theoretical constructs involved. The model demonstrated a good fit (see Table 3) given that the Satorra-Bentler Chi Square, even after a Bonferroni-correction, remained non-significant (see Table 3). Furthermore, other fit indices indicated a good fit. Discriminant validity was established by showing that fixing the correlations between the constructs at 1 would significantly lower the model fit in all cases (Anderson and Gerbing, 1988). Furthermore, the significant factor loading and the average variance extracted exceeding 0.5 indicated convergent validity. Composite reliability was assessed by calculating Weighted Ω (Bacon et al., 1995) which is considered to better reflect the true reliability than Cronbach’s α (Schmitt, 1996).

**TABLE 3:** Latent variable measurement models.

	N	SB X2	df	RMSEA	CFI	NNFI	GFI
M0 measurement model	262	70.31*	51	0.038	0.994	0.992	0.991
M1 measurement model	262	49.16	41	0.028	0.997	0.996	0.993

* *p* < 0.05.

### Analysis

The hypotheses will be tested by means of multiple moderated regression (Baron and Kenny, 1986). This enables the conditional nature of effects to be assessed. Variables were not standardized or centralized as this would not resolve “imaginary multicollinearity” issues created by the nature of moderator analysis (Dawson, 2014; Hayes, 2014). Moderation effects were tested for based on regular standard errors (SAS PROC GLM) and robust standard errors (SAS PROC REG) (White, 1980) in order to account for possible heteroscedasticity (Wooldridge, 2009). The effects were plotted to ease interpretation using SAS ODS and SAS PROC PLM. Although the data can at best be considered as pseudo-metrics, the analysis is based on OLS regression as this provides more meaningful interpretations (Allison, 1999). Given that the hypotheses are directional, they were subjected to one-sided tests.

## Results

First, some descriptive statistics and the correlation matrix are presented and discussed. Subsequently, the results of moderation tests are provided, followed by the treatment checks. Finally, some additional analysis in terms of robustness checks is provided.

### Descriptives

Analyses of the descriptives (see Tables 1, 4) indicate that, on average, attractiveness is positive, with an average score of 3.63 across the entire sample. Similarly, the levels of public service motivation are rather high with a mean score of 4.15. This is not that surprising given that those who were targeted in this sample were those who, either by means of education or experience, were likely candidates for working in a municipality. Obtaining a degree in a field that usually sorts into a public service occupation (such as public law or social sciences) has been shown to lead to higher scores on this variable than degrees in other fields (Vandenabeele, 2008a, 2011) and respondents retain these relatively high scores even if their public service motivation drops due to a reality shock when entering public service (Kjeldsen and Jacobsen, 2012). The third factor, PO-fit, had the lowest mean score of 3.44.

**TABLE 4:** Correlation table.

		Mean	SD	1.	2.	3.	4.	5.
1.	Attractiveness	3.63	0.70					
2.	PO-fit	3.43	0.65	0.559***				
3.	PSM	4.15	0.64	0.291***	0.219**			
4.	Public	0.38	0.49	–0.115	0.000	0.004		
5.	Private	0.30	0.46	0.026	0.088	–0.115	−0.510***	
6.	Control	0.32	0.47	0.093	–0.087	0.109	−0.535***	−0.454***

** *p* < 0.01; *** *p* < 0.001.

The correlation table indicates that public service motivation, PO-fit, and attractiveness are all positively correlated with each other (see Table 5).

**TABLE 5:** Linear regression analysis^*a*^ of Attractiveness and Person–Organization fit (regular and robust standard errors).

	Attractiveness				Person–Organization fit			
	β		β		β		β	
	SE	SE White	SE	SE White	SE	SE White	SE	SE White
Public	−0.167		−1.540	*/*	0.104		−2.516	***/***
	0.116	0.115	0.819	0.755	0.109	0.110	0.752	0.692
Private	0.001		−0.995		0.219−/	*	−0.797	
	0.123	0.121	0.779	0.704	0.116	0.109	0.715	0.608
PSM	0.320	***/***	0.128		0.240	**/**	−0.045	
	0.077	0.073	0.137	0.126	0.072	0.076	0.126	0.101
Public X PSM			0.326	*/*			0.625	***/***
			0.193	0.186			0.177	0.167
Private X PSM			0.237				0.237	
			0.185	0.169			0.170	0.148
N	192		192		192		192	
F	6.82	***	4.73	***	4.40	**	5.33	***
R^2^	0.098		0.113		0.066		0.125	
Adj. R^2^	0.084		0.089		0.051		0.102	

* *p* < 0.05; ** *p* < 0.01; *** *p* < 0.001. ^*a*^One-sided testing.

### Testing the Hypotheses

Before testing the hypotheses, a randomization check on the survey-experiment was carried out. Tests for age, *F*(2, 180) = 1.77, *p* = 0.174, and gender, χ^2^(df = 2) = 4.00, *p* = 0.135, indicate that the respondents were distributed randomly across the three conditions.

**FIGURE 1:**
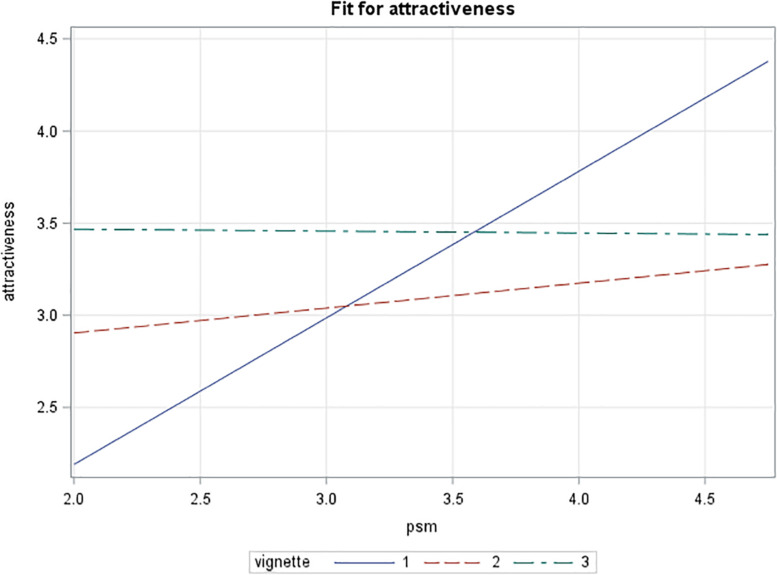
Interaction effect of vignette type and public service motivation on attractiveness (full sample).

A multiple moderated regression was performed to test the hypotheses. In Table 5, four regression models are presented. First, a main effects and then a moderator model for attractiveness were tested. Both models indicate significant omnibus tests, with *F*(3,188) = 6. 82, *p* = 0.0002 and *F*(5,186) = 4.73, *p* = 0.0004 respectively. For the main effects model, *R*^2^ = 0.098 where for the interaction model *R*^2^ = 0.113, indicating a small to moderate effect size (Cohen, 1992). With a significant interaction effect, the effect size should be understood in terms of a system since the interpretations of main effect and interaction effect are meaningless in isolation (Aiken and West, 1991). The *R*^2^ value of 0.113 corresponds to an *R* of 0.336, which slightly exceeds the threshold of 0.30 for a moderate effect. An interaction effect between the public vignette and public service motivation was found at a significance level of *p* < 0.05. Both the regular and robust standard errors indicate similar significant effect sizes, although in the robust case the White errors are marginally smaller (indicating some heteroskedasticity) leading to lower p-values. From a plot of the effect (see Figure 1), it can be observed that for potential applicants with low levels of public service motivation, the message including public values (vignette 1) is the least attractive, whereas the private values message (vignette 2) is somewhat more attractive and the control version (vignette 3) is the most attractive. All the vignettes are more attractive, and all to a similar level, to those with high levels of public service motivation (illustrating the main effect of public service motivation).

**FIGURE 2:**
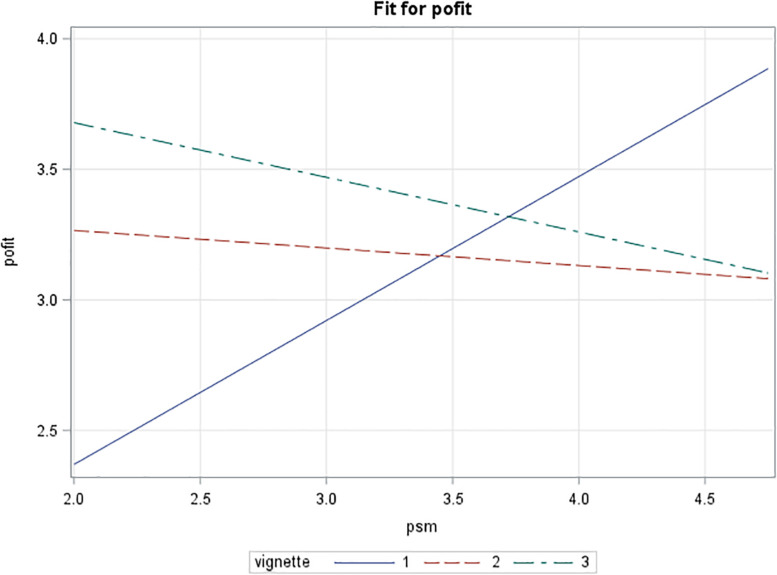
Interaction effect of vignette type and public service motivation on person–organization fit (full sample).

Next, a main effects and an interaction model for person–organization fit were tested. Again, both models illustrate significant omnibus tests, with *F*(3,188) = 4.40, *p* = 0.0051 and *F*(5,186) = 5.33, *p* = 0.0001, respectively. For the main effects model, *R*^2^ = 0.066, and for the interaction model *R* = 0.125, again indicating a small to moderate effect size (Cohen, 1992). Compared to the public service motivation models, the difference here between the main effects model and the interaction model was larger in terms of explained variance, indicating a stronger interaction effect. The main effects model showed a positive relationship between public service motivation and person–organization fit for those receiving the public or the private vales-based recruitment messages. The private values message had a positive effect on person–organization fit based on a robust standard error test. As with public service motivation, an interaction effect between the public vignette and public service motivation was found at the *p* < 0.05 level. Here, both regular and robust standard errors suggest similar significant effects. The plotting of the effects (see Figure 2) illustrates that, for those with low levels of public service motivation, the message including public values (vignette 1) again creates the worst person–organization fit, whereas both the private values and the neutral versions (vignettes 2 and 3) result in a better fit. When levels of public service motivation are high, both the public and private values messages create a good fit with the organization.

### Further Analyses: Exploring More Deeply

A puzzling finding is that, although the main effects of the vignettes do not suggest that attractiveness has a significant effect, with low levels of public service motivation both the private and public values versions of the vignette seem to be substantially less attractive than a version where no values are highlighted. Higher levels of public service motivation can increase the attractiveness of the value-based vignettes to a level that is similar to that of the “neutral” vignette (the control group). Despite the observed moderation effects, this is not fully in line with what theory suggests and what is claimed in practice. That is, one would expect referring to public values to create a competitive advantage, something extra. Instead, what we see is that it barely suffices to keep up. Considering person–organization fit, Figure 2 shows that there is some competitive advantage in matching an individual’s values to public service motivation, but not overwhelmingly. This points more toward a buffering or selection effect than a genuine competitive advantage.

The PO-fit analysis however indicates that the effect of the public condition (vignette 1) in combination with PSM is in line with what was hypothesized. This means that the previously identified lack of attractiveness needs to be rooted elsewhere, that there is other information that reduces the attractiveness, despite this conditional effect for PO-fit (which indicates that it is an important determinant of attractiveness). This has also been illustrated by Asseburg and Homberg (2020), who found that the attractiveness of public sector employment does not only depend on value fit. However, the more general information is similar in all three vignettes, and thus cannot explain this observation. In considering employer branding, it has been stated that “would-be employees rarely have perfect information about a prospective employers” (Wilden et al., 2010, p. 59). In our study, this knowledge is not equal across all respondents. Given that a large proportion of the respondents have work experience within the public sector, it may be that they have information that people with no experience in the public sector lack. As branding is linked to signaling theory (Spence, 1973), apart from the explicit signals provided in the recruitment message, some groups of prospective employees may receive implicit signals from previous, message-unrelated, experiences. In other words, they may have inside information on how a municipality works and base their attitude toward the organization on this as well on the explicit signals. If these implicit signals are negative, it may reduce attractiveness, regardless of the positive explicit signals received. Such information asymmetry may be behind our findings.

Therefore, although the sample includes only have a small group of people that have worked outside the public sector and have private sector experience, some exploratory analyses will be performed to see whether work experience – as an implicit signal – plays a role. The subsample with respondents who are currently not employed and have no work experience in the public sector is very limited. Only 37 of the respondents meet this criterion (15 given the public vignette, 12 the private vignette, and 10 the control vignette). Hence, and also because this exercise is not testing theory but rather exploring potential new paths, significance testing is not a key focus. Rather, the prime objective is to identify response patterns and investigate effect sizes in a explorative fashion to provide information for future research.

Despite the small sample sizes and the lack of a model showing statistically significant effects (Table 6), there is an indication that public service motivation has an interaction effect on the relationship between the public vignette and attractiveness. This effect appears more significant based on robust standard errors (although robust standard error may produce biased results with small sample sizes) (Wooldridge, 2009). A similar effect is found for this combination when looking at person–organization fit. That significant effects are found indicates that effect sizes are substantial – something that is supported by the large *R*^2^ values.

**TABLE 6:** Linear regression analysis ^a^ of Attractiveness and Person–Organization fit for private sector employees (regular and robust standard errors).

	Attractiveness		Person–Organization fit	
	β		β	
	SE	SE White	SE	SE White
Public	−2.888	*/***	−2.831	*/***
	1.473	0.657	1.496	0.615
Private	−0.854		−0.697	
	1.284	0.732	1.304	0.713
PSM	−0.010		−0.209	
	0.278	0.141	0.282	0.084
Public X PSM	0.806	*/***	0.761	*/***
	0.397	0.191	0.403	0.176
Private X PSM	0.146		0.142	
	0.351	0.213	0.356	0.198
N	37		37	
F	2.32		0.95	
R^2^	0.273		0.133	
Adj. R^2^	0.155		−0.007	

* *p* < 0.05; ** *p* < 0.01; *** *p* < 0.001. ^*a*^One-sided testing.

**FIGURE 3:**
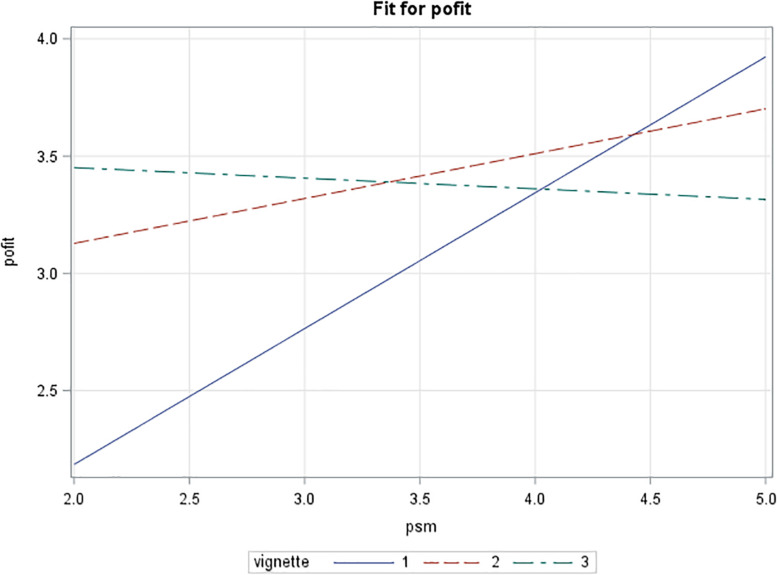
Interaction effect of vignette type and public service motivation on attractiveness (private sector sample).

**FIGURE 4:**
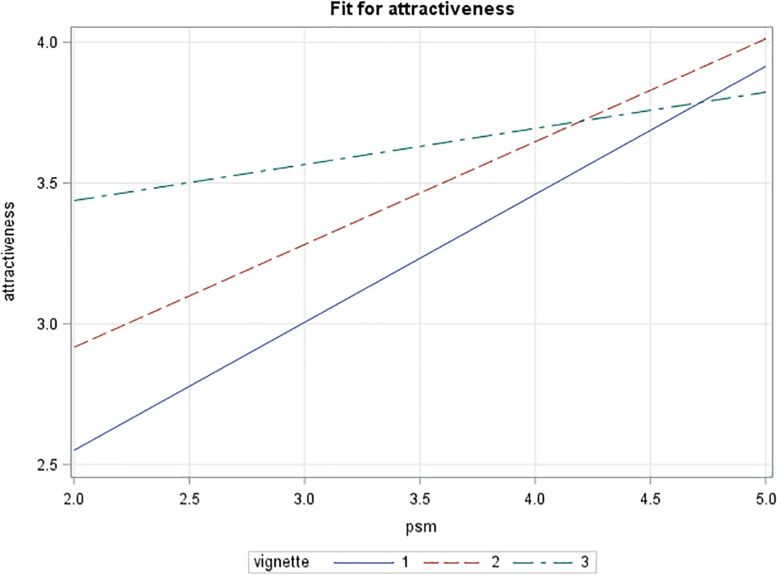
Interaction effect of vignette type and public service motivation on person–organization fit (private sector sample).

When plotted, the patterns shown are as expected from theory: with low levels of PSM leading to low scores for attractiveness of the public conditions, and the neutral control condition being more attractive (see Figure 3). Further, high scores for public service motivation link with a strong attraction for the public conditions and less for the neutral conditions. With both PSM levels, the private conditions produce a level of attraction between the other two scenarios. A similar pattern is found for person–organization fit (see Figure 4). More importantly, the public vignette seems to create a competitive advantage rendering the vacancy much more attractive for highly public service motivated prospective employees.

## Discussion

The analysis of the results from the survey experiment as described above has been insightful in many ways. First, in terms of the moderating analysis for attractiveness, only H1A has been corroborated by our total sample, despite finding a significant moderating term. This is reflected in the observation that a match between public service motivation and the public vignette can create a level of attractiveness, but it does not create a competitive advantage over a neutral condition. As such, H1B has to be rejected. Third, when carrying out a similar analysis in terms of person–organization fit, similar patterns can be found, with H2A being supported and H2B rejected.

These findings to some extent support existing theory on public service motivation and organizational attractiveness, supporting the causal model of person–organization fit in terms of public service motivation and environmental cues related to the organizational context. As such, it not only provides support for the idea of an institutional logic of appropriateness within an institution (March and Olsen, 1989), it also supports the idea that there could be an anticipatory logic of appropriateness that causes people to select particular organizations. As such, public service motivation theory fully aligns with institutional theory, even extending its scope. In this sense, it illustrates the early contention that institutions not only delimit but also enable (Giddens, 1984). This is a finding that may have value for other types of prosocial motivation. To our knowledge, institutional theory has scarcely been integrated with prosocial motivation in general. However, the conditional nature of the effect of public service motivation, depending as it does on the institutional environment and its values, could further our knowledge on a broader concept of prosocial motivation. However, future research needs to address this before one can draw firm conclusions.

Furthermore, the rejection of hypotheses H1B and H2B calls for further development of the institutional foundations upon which public service motivation theory rests. The exploratory analysis of potential job seekers outside the public sector may offer some valuable insights. Supplementing public service motivation theory with insights from signaling theory, including explicit and implicit signals that potentially create information asymmetry in terms of branding (Wilden et al., 2010), may prove a valuable direction to further develop public service motivation theory. However, it must again be stressed that these findings are exploratory and therefore require solid further research.

For practice, these findings illustrate that including public service motivation in recruitment messages is beneficial, but that it is not a silver bullet for solving recruitment issues. In fact, the current results suggest that recruitment messages should cater for the needs of whichever parts of the public are being addressed. For an audience that has public sector experience, the implicit messaging may also be very important even if public service motivation is not addressed explicitly. Regardless of the message, the mere fact that a public organization advertises enables the characteristics of the organization and associated jobs to be implicitly related to that organization. Furthermore, in such a situation, relying on public values and public service motivation could act as a buffer to discourage non-public service motivated applicants. This could be beneficial since public service motivation has been associated with numerous outcomes that are deemed positive (Ritz et al., 2016). In this scenario, recruitment messages create a sorting effect and may operate as a self-selection instrument, rather than purely as a recruitment instrument.

Despite these findings, there are some limitations that should be considered when interpreting these results. First, in terms of the nature of the sample, the current sample consists mainly of individuals who are oriented toward public employment. This is a strength in terms of external validity, as this is the population from which municipalities draw the bulk of their recruits. However, at the same time, this has a downside in that it means there is substantial implicit knowledge that is not captured in the model, and may even cause plateauing effects that may explain the failure to identify expected relationships due to a low variance in implicit signals. Second, and related to this, the sample included only a few that did not have public sector work experience and so not all effects could be tested in a satisfactory manner. Third, the survey-experiment design with vignettes only captures behavioral intentions, not actual behavior. Nevertheless, this does not weigh against the cost in terms of setting up a field experiment and the potential associated opportunity costs when losing out on prospective employees due to variation in recruitment messages in such a field experiment (which may cause some good people to not apply). Furthermore, the design using survey experiments, with written vignettes as the manipulation, limits manipulation checks as no tasks are performed meaning that we had to rely on indirect measures such as exposure and recall information to check the data quality. Finally, the current analysis excludes other fit perspectives such as person–job fit. However, given the strong association between the various fit concepts found empirically (Van Loon et al., 2017), it is unlikely that including others would substantially alter the findings.

Given these limitations, future research should replicate this experiment to re-assess the findings with both similar and different samples (in particular including more people with private sector experience). Further, other implicit signals may also be available that could be tested for their influence on attractiveness and their relationship withpublic service motivation. Furthermore, other types of prosocial motivation could be included to see whether they also have similar effects. More complex experiments could be established (such as conjoint experiments, varying in multiple aspects of the offer) to assess the effectiveness of various types of job offer.

## Data Availability Statement

The original contributions presented in the study are included in the article/Supplementary Material, further inquiries can be directed to the corresponding author.

## Ethics Statement

Ethical review and approval was not required for the study on human participants in accordance with the local legislation and institutional requirements. The patients/participants provided their written informed consent to participate in this study.

## Author Contributions

WV analyzed the data, wrote the manuscript, and contributed to the design. SJ contributed to the design and collected the data. Both authors contributed to the article and approved the submitted version.

## Conflict of Interest

The authors declare that the research was conducted in the absence of any commercial or financial relationships that could be construed as a potential conflict of interest.

## Annex: Overview of Vignettes Used in Survey (Value Cues in Bold; Originally in Dutch and Available Upon Request)

### Vignette Public Values Condition

Strategic advisor

Do you want to contribute to **solving societal issues**? Do you think it is important that **citizens** can rely on **a stable provision of public services**? Do you address policy challenges straightforward? Then we are looking for you

This is what you do

You know what is going on in society and what challenges we face. You can reflect on what it means for our municipality, make the right connections and translates this in in clear language to plans, ideas or policies that are effective. You can do this for challenges within our municipal boundaries, but also for those outside the municipality at a regional level. It does not matter what the particular topic is, as you are multi-disciplinary and you enjoy this general perspective.

You are …

– You are educated at a bachelor or master degree level
– You enjoy solving complicated puzzles in various domains
– When developing policy, you think it is important **to take into account the interests of all our citizens**
– You are a keen on developing an interactive process and you know your role within this process; you know how to get the right results
– **You are prepared to be completely at the service of society**

We offer

You can **contribute to a community that addresses the needs of as many citizens as possible.** You have excellent employment conditions and you will enjoy many benefits that will help you to develop to further your career prospects. Depending on experience and education, the salary will be maximum 4533 euro before taxes (scale 11) in a 36 hour workweek.

We are

We are a modern municipality with an open atmosphere. Our culture is very informal, we operate integral and interactive. Development is permanent and mandatory: we know what is going on in the municipality and we respond to that. **The ultimate goal is the public interest**.

You will work with the department of strategy and governance. This department is is situated at the start of any policy-cycle. At this department are working, apart from other strategic advisors, administrative advisors and support staff.

### Vignette Private Values Condition

Strategic advisor

Do you want to contribute to **innovative solutions**? Do you think it is important to operate **professionally and reliably**? Do you address policy challenges straightforward? Then we are looking for you

This is what you do

You know what is going on in society and what challenges we face. You can reflect on what it means for our municipality, make the right connections and translates this in in clear language to plans, ideas or policies that are effective. You can do this for challenges within our municipal boundaries, but also for those outside the municipality at a regional level. It does not matter what the particular topic is, as you are multi-disciplinary and you enjoy this general perspective.

You are …

– You are educated at a bachelor or master degree level
– You enjoy solving complicated puzzles in various domains
– When developing policy, you think it is important to **operate efficiently**
– You are a keen on developing an interactive process and you know your role within this process; you know how to get the right results
– **Innovation is your trademark, as well in thinking as acting**

We offer

You can contribute to an **innovative and professional** policy at the municipal level. You have excellent employment conditions and you will enjoy many benefits that will help you to develop to further your career prospects. Depending on experience and education, the salary will be maximum 4533 euro before taxes (scale 11) in a 36 hour workweek.

We are

We are a modern municipality with an open atmosphere. Our culture is very informal, we operate integral and interactive. Development is permanent and mandatory: we know what is going on in the municipality and we respond to that. **The ultimate goal is the efficiency and reliability.**

You will work with the department of strategy and governance. This department is is situated at the start of any policy-cycle. At this department are working, apart from other strategic advisors, administrative advisors and support staff.

### Vignette Control Condition

Strategic advisor

Do you address policy challenges straightforward? Then we are looking for you

This is what you do

You know what is going on in society and what challenges we face. You can reflect on what it means for our municipality, make the right connections and translates this in in clear language to plans, ideas or policies that are effective. You can do this for challenges within our municipal boundaries, but also for those outside the municipality at a regional level. It does not matter what the particular topic is, as you are multi-disciplinary and you enjoy this general perspective.

You are …

– You are educated at a bachelor or master degree level
– You enjoy solving complicated puzzles in various domains
– You are a keen on developing an interactive process and you know your role within this process; you know how to get the right results

We offer

You have excellent employment conditions and you will enjoy many benefits that will help you to develop to further your career prospects. Depending on experience and education, the salary will be maximum 4533 euro before taxes (scale 11) in a 36 hour workweek.

We are

We are a modern municipality with an open atmosphere. Our culture is very informal, we operate integral and interactive. Development is permanent and mandatory: we know what is going on in the municipality and we respond to that.

You will work with the department of strategy and governance. This department is is situated at the start of any policy-cycle. At this department are working, apart from other strategic advisors, administrative advisors and support staff.
